# Avoidable hospitalizations for ambulatory care sensitive conditions in children under five years in Ecuador, 2000-2023

**DOI:** 10.1590/0102-311XEN098425

**Published:** 2025-12-01

**Authors:** Carolina Buñay-Morocho, Pablo Álvarez, Daniel Zurita, Miguel Martin, Philip Cooper, Natalia Romero-Sandoval, Monsermin Gualán

**Affiliations:** 1 Universidad Internacional del Ecuador, Quito, Ecuador.; 2 Universidad Autónoma de Barcelona, Bracelona, España.

**Keywords:** Ambulatory Care Sensitive Conditions, Primary Healthcare, Quality of Health Care, Condiciones Sensibles a la Atención Ambulatoria, Atención Primaria de Salud, Calidad de la Atención de Salud, Condições Sensíveis à Atenção Primária, Atenção Primária à Saúde, Qualidade da Assistência à Saúde

## Abstract

Avoidable hospitalizations due to ambulatory care sensitive conditions (ACSC) are an indirect indicator of primary health care quality and effectiveness of care coordination. This study aims to analyze the proportion and trends of hospital discharges for ACSCs (2000-2023) among children under five years, project rates through 2026, and compare standardized rates across cantons. We conducted an ecologic time-series analysis using Ecuador’s national hospital discharge data for 20 ACSCs, as defined by the Pan-American Health Organization. Annual percentage changes were estimated using Joinpoint regression, and forecasts were generated with the Prophet package in R. Standardized morbidity ratios (SMRs) were used to compare rates across 221 cantons, based on Ecuador’s population from the 2001, 2010, and 2022 censuses. Between 2000 and 2023, ACSCs accounted for 26.6% of all hospital discharges. The overall average of annual percent change increased by 2%, and by 6.8%, 6.4%, and 4.2% for respiratory diseases, urinary and skin infections, respectively. Gastrointestinal diseases declined by 1.9% annually. Significant changes in ACSC trends were observed during the following periods: 2000-2007; 2018-2021; and 2021-2023. No significant change occurred from 2008 to 2018. Projections indicated that ACSCs may still represent 20.3% of hospital discharges by 2026. Moreover, 5.4% of cantons consistently exceeded expected SMRs across all three census years analyzed. The rising ACSC rates during the early 2000s, marked by economic structural adjustment and limited public healthcare investment, contrasts with the decline observed during the COVID-19 pandemic. These findings underscore the need to strengthen primary care and public health planning.

## Abstract (es)

Las hospitalizaciones evitables debido a condiciones sensibles a la atención primaria (CSAP) son un indicador indirecto de la calidad de la atención primaria y de la eficacia en la coordinación de la atención. Nuestro objetivo era analizar la proporción y las tendencias del alta hospitalaria por CSAP (2000-2023) entre niños menores de cinco años, proyectar las tasas hasta 2026 y comparar las tasas estandarizadas entre los distintos cantones. Realizamos un análisis ecológico de series temporales utilizando los datos nacionales de alta hospitalaria de Ecuador para 20 CSAP de la Organización Panamericana de la Salud. Los cambios porcentuales anuales se estimaron utilizando la regresión Joinpoint, y las previsiones utilizando el paquete Prophet en R. Las tasas de morbilidad estandarizadas (TME) compararon las tasas en 221 cantones utilizando la población de Ecuador de los censos de 2001, 2010 y 2022. Entre 2000 y 2023, las CSAP representaron el 26,6 % de todas las altas hospitalarias. El cambio porcentual medio anual aumentó un 2% en general y un 6,8%, 6,4% y 4,2% en el caso de las enfermedades respiratorias y las infecciones urinarias y cutáneas, respectivamente. Las enfermedades gastrointestinales disminuyeron un 1,9% al año. Se observaron cambios significativos en las tendencias de CSAP para los períodos: 2000 a 2007; 2018 a 2021; y 2021 a 2023. No se produjeron cambios significativos entre 2008 y 2018. Las proyecciones indicaban que las CSAP podrían seguir representando el 20,3% de la alta hospitalaria en 2026. El 5,4% de los cantones superó sistemáticamente las TME previstas durante los tres años de censo incluidos en el período de observación. El aumento de las tasas de CSAP durante el período de ajuste estructural económico a principios de la década de 2000 se caracterizó por una inversión limitada en salud pública y contrastó con el descenso observado durante la pandemia de COVID-19. Es fundamental reforzar la atención primaria y la planificación de la salud pública.

## Abstract (pt)

As hospitalizações evitáveis ​​devido a condições sensíveis à atenção primária (CSAP) são um indicador indireto da qualidade da atenção primária à saúde e da eficácia da coordenação do cuidado. Nosso objetivo foi analisar a proporção e as tendências de altas hospitalares por CSAP (2000-2023) entre crianças menores de cinco anos, projetar taxas para o ano 2026 e comparar taxas padronizadas entre cantões. Fizemos uma análise ecológica de séries temporais usando dados nacionais de altas hospitalares do Equador para 20 CSAP da Organização Pan-Americana da Saúde. As variações percentuais anuais foram estimadas usando regressão Joinpoint e as previsões usando o pacote Prophet em R. As razões de morbidade padronizadas (RMP) compararam as taxas entre 221 cantões usando a população do Equador dos censos de 2001, 2010 e 2022. Entre 2000 e 2023, as CSAP representaram 26,6% de todas as altas hospitalares. A variação percentual média anual aumentou 2% no geral e 6,8%, 6,4% e 4,2% para doenças respiratórias, infecções urinárias e de pele, respectivamente. As doenças gastrointestinais diminuíram 1,9% ao ano. Mudanças significativas nas tendências de CSAP foram observadas nos períodos: 2000 a 2007; 2018 a 2021; e 2021 a 2023. Não houve mudanças significativas entre 2008 e 2018. As projeções indicaram que as CSAP ainda podem representar 20,3% da população em alta hospitalar em 2026. Além disso, 5,4% dos cantões excederam consistentemente as RMP esperadas durante todos os três anos censitários incluídos no período de observação. O aumento das taxas de CSAP durante o período de ajuste estrutural econômico no início dos anos 2000 foi marcado por investimentos limitados em Saúde Pública e contrastou com o declínio observado durante a pandemia de COVID-19. O fortalecimento da atenção primária e do planejamento da Saúde Pública é criticamente necessário.

## Introduction

Primary health care (PHC) is a comprehensive approach that aligns health services with the needs of individuals, families, and communities, ensuring timely and equitable access to health ^1^. An indirect measure of PHC quality and accessibility is the analysis of avoidable hospitalizations due to ambulatory care sensitive conditions (ACSC), which can be prevented with effective PHC interventions ^2^ ^,^ ^3^ ^,^ ^4^. The concept, first introduced in the 1980s ^5^, was formally recognized as an indicator by the U.S. Institute of Medicine in 1993 ^6^ ^,^ ^7^. In 2014, the Pan-American Health Organization (PAHO) categorized 20 condition groups as ACSCs, using codes from the 10th revision of the International Classification of Diseases (ICD-10) ^8^.

The evaluation of ACSC is key for care coordination and health system decision-making, guiding efforts to strengthen PHC and improve its integrated networks ^9^ ^,^ ^10^. Rising avoidable hospitalizations due to ACSC may reflect limitations in a country’s PHC system ^10^ ^,^ ^11^. Among children under five years of age, these hospitalizations are associated with poorer health outcomes, increased mortality risk, and significant tensions on the functioning of the first level of care ^12^.

A report on ACSCs in six Latin American countries identified Ecuador as having the second highest ACSC rate across all age groups in 2012 ^13^. In Brazil, the decrease of ACSC for gastroenteritis was attributed to the implementation and expansion of PHC programs ^11^ ^,^ ^14^. In this context, monitoring ACSC is crucial for optimizing resource allocation and setting healthcare priorities ^9^ ^,^ ^10^ ^,^ ^11^.

No published studies have examined trends in ACSC-related hospitalizations among children under five years of age in Ecuador. We aim to analyze the proportion of avoidable hospitalizations due to ACSC among children under five years of age (2000-2023) by PAHO-defined condition groups, assess temporal trends, estimate annual percentage changes, project rates to 2026, and compare standardized rates across cantons using census-year populations.

## Methods

This ecological time-series study used data from the Ecuadorian National Register of Hospital Discharges, provided by the Ecuadorian National Institute of Statistics and Census ^15^. Annual records were merged using canton codes as identifiers. Avoidable hospitalizations due to ACSC were identified using ICD-10 codes defined by PAHO ^8^. Respiratory-related ACSC were grouped into a single “respiratory diseases” category.

Crude hospitalization rates per 10,000 children under five years of age were calculated using annual population estimates from 2022 ^15^. Joinpoint regression was applied to assess temporal trends in overall and condition-specific rates.

Forecasts for 2026 were generated using the *Prophet* library (version 1.0) in R (http://www.r-project.org), which applies an additive time-series model. Default settings were used to automatically detect changepoints, and projections extended three years beyond the last observed data point (2023).

Standardized morbidity rates (SMRs) were calculated using indirect standardization by sex and age (< 1, 1-2, and 3-4 years), based on national census data from 2001, 2010, and 2022, and reported with 95% confidence intervals (95%CI).

Data management and statistical analyses were performed using RStudio (https://rstudio.com/) and QGIS (https://qgis.org/en/site/). Joinpoint regression was conducted with Joinpoint software, version 5.3.0 (https://surveillance.cancer.gov/joinpoint/).

## Results

Ecuador recorded 2,890,251 hospital discharges among children under five years of age between 2000 and 2023, of which 768,766 (26.6%; 95%CI: 26.53, 26.66) were avoidable hospitalizations due to ACSC (Table 1). The most frequent avoidable hospitalizations due to ACSC were gastrointestinal infections and complications (55.2%; 95%CI: 55.03, 55.36), respiratory diseases (24.84%; 95%CI: 24.73, 24.95), kidney and urinary tract infections (6.72%; 95%CI: 6.66, 6.77), and skin and subcutaneous tissue infections (6.12%; 95%CI: 6.07, 6.18).

**Table 1:** Percentage of avoidable hospitalizations due to ambulatory care sensitive conditions (ACSC) among children under five years of age in Ecuador, 2000-2023.

Year	Hospitalizations by ACSC	Total hospitalizations	% (95%CI)
2000	23,025	77,807	29.59 (29.21, 29.98)
2001	24,820	78,712	31.53 (31.14, 31.93)
2002	25,242	84,280	29.95 (29.58, 30.32)
2003	28,252	91,603	30.84 (30.48, 31.20)
2004	30,363	99,632	30.48 (30.13, 30.82)
2005	32,227	103,883	31.02 (30.69, 31.36)
2006	36,143	112,751	32.06 (31.73, 32.39)
2007	39,946	116,377	34.32 (33.99, 34.66)
2008	37,229	128,181	29.04 (28.75, 29.34)
2009	33,953	131,872	25.75 (25.47, 26.02)
2010	39,073	145,544	26.85 (26.58, 27.11)
2011	34,602	142,371	24.30 (24.05, 24.56)
2012	34,823	143,621	24.25 (23.99, 24.50)
2013	36,712	145,846	25.17 (24.92, 25.43)
2014	37,965	149,470	25.40 (25.15, 25.66)
2015	36,307	142,990	25.39 (25.13, 25.65)
2016	39,458	144,389	27.33 (27.06, 27.60)
2017	35,743	138,821	25.75 (25.48, 26.02)
2018	33,289	134,169	24.81 (24.55, 25.08)
2019	35,961	138,092	26.04 (25.77, 26.31)
2020	15,973	88,330	18.08 (17.81, 18.37)
2021	19,814	95,816	20.68 (20.39, 20.97)
2022	27,495	122,159	22.51 (22.24, 22.78)
2023	30,351	130,244	23.30 (23.04, 23.57)
2000-2023	768,766	2,886,960	26.63 (26.53, 26.66)

95%CI: 95% confidence interval.

**Figure 1:**
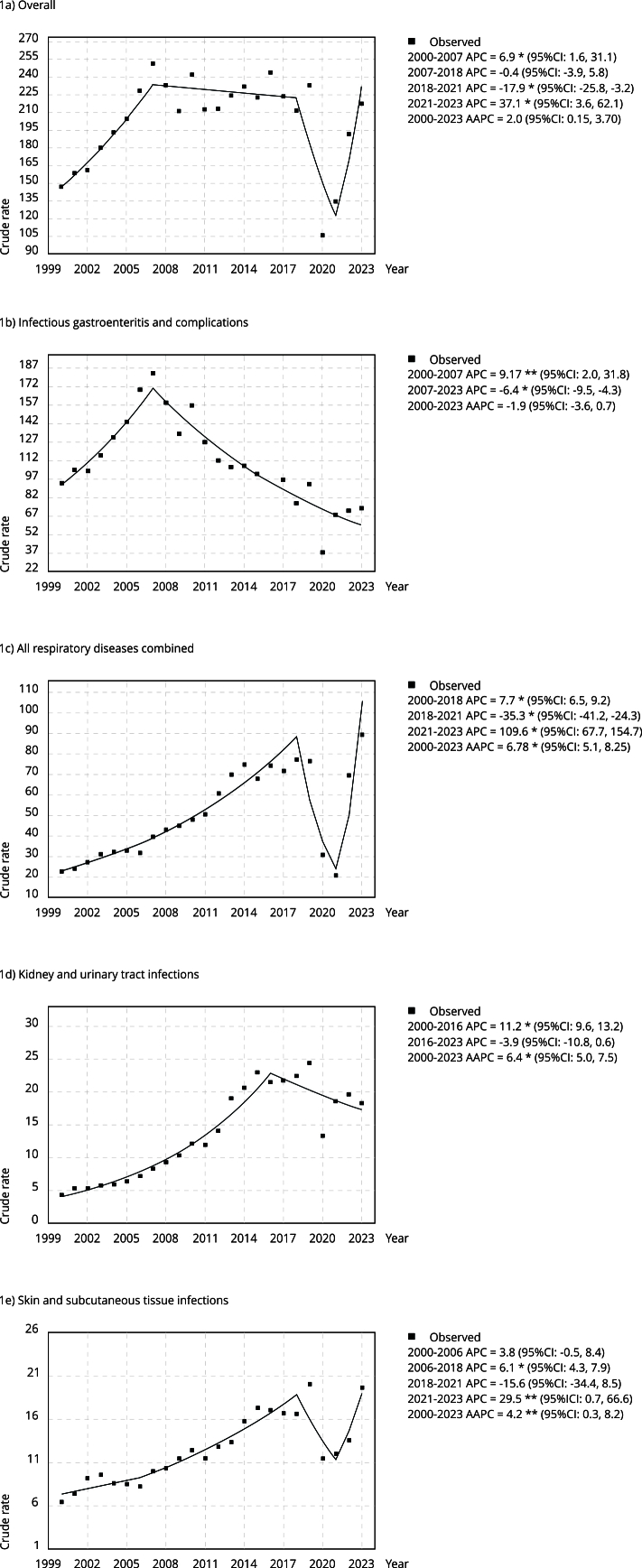
Joinpoint analysis of crude hospital discharge rates due to ambulatory care sensitive conditions (ACSC) among children under five years of age. Ecuador, 2000-2023.

Figure 1 summarizes the trends for overall ACSCs and for the four most common ICD-10 codes. Rates increased annually by 6.86% from 2000 to 2007 (p < 0.01), declined by -0.44% from 2007 to 2018 (p > 0.05), decreased by -17.89% from 2018 to 2021 (p < 0.01), and then rose by 37.05% between 2021 and 2023 (p < 0.01). Overall, hospitalizations exhibited a positive average of annual percent change (AAPC) of 2.01% (95%CI: 0.15, 3.67). AAPCs for the most common ACSC groups ranged from 4.18% (95%CI: 0.29, 8.22) for skin and subcutaneous tissue infections to 6.78% (95%CI: 5.11, 8.25) for respiratory diseases, while gastrointestinal infections and complications declined by -1.89% (95%CI: -3.61, 0.68).

95%CI: 95% confidence interval; AAPC: average of annual percent change; APC: annual percent change. * p < 0.01; ** p < 0.05.

**Figure 2:**
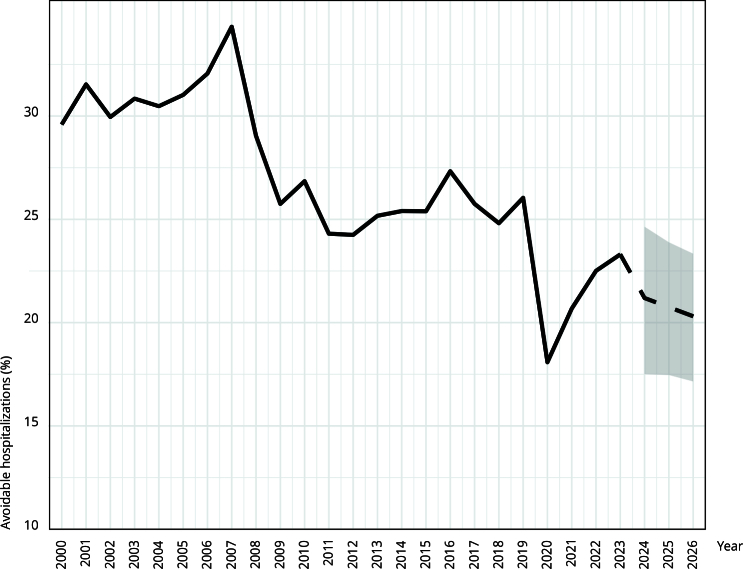
Percentage of avoidable hospitalizations due to ambulatory care sensitive conditions (ACSC) among children under five years in Ecuador, 2000-2023, and projections to 2026 with 95% confidence intervals.

Note: projections are represented by dashed lines.

Figure 2 illustrates the observed proportion of avoidable hospitalizations due to ACSC among children under five years of age during the study period, as well as the projected trend through 2026. Under the lower-bound estimate, the projected proportion reached 20.3% (95%CI: 17.1, 23.6).

**Figure 3:**
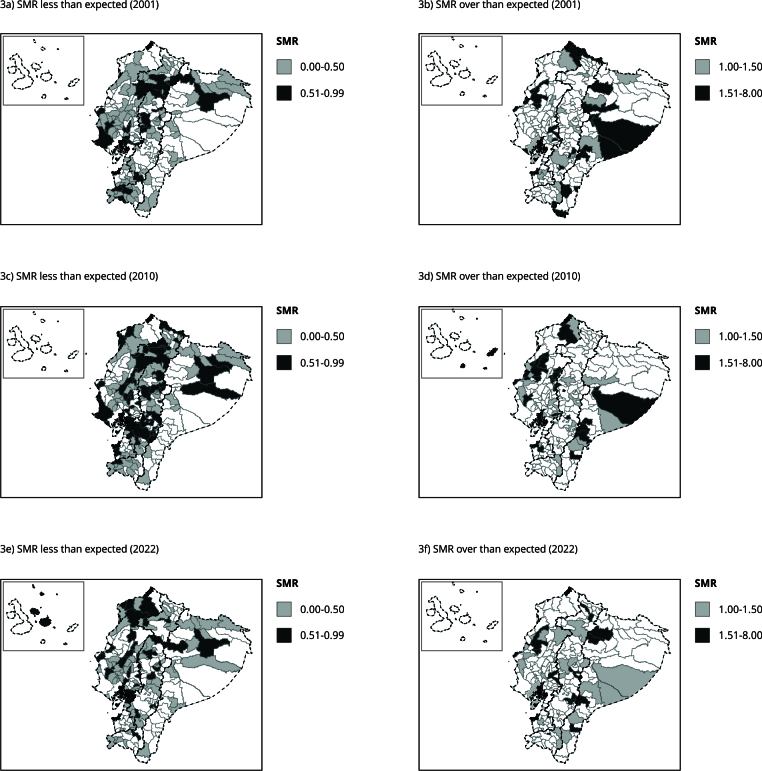
Standardized morbidity ratio (SMR) of hospital discharges for ambulatory care sensitive conditions (ACSC) among children under five years by Ecuadorian canton in 2001, 2010, and 2022.

Figure 3 shows SMRs for the three census years, with 41 (18.55%), 38 (17.19%), and 36 (16.29%) cantons exceeding expected hospitalization rates in 2001, 2010, and 2022, respectively. Meanwhile, 116 (52.49%), 130 (58.82%), and 110 (49.77%) cantons had lower-than-expected values. Across the study period, 12 cantons (5.43%) consistently recorded higher-than-expected rates.

## Discussion

Previous studies in Ecuador reported that ACSCs accounted for 17% of all hospitalizations across all age groups between 2001 and 2010 ^13^ ^,^ ^16^, with no prior estimates for children under five years of age. In this analysis, we focused on this age group and found that ACSCs represented 26.6% of all hospital discharges.

We identified a total of 19 ACSC condition groups, with no recorded discharges for heart failure. Gastrointestinal infections and complications were the leading causes, accounting for over 50% of avoidable hospitalizations due to ACSC early in the study period but declining to less than 40% in recent years. This trend is consistent with a 2006 Brazilian study, which reported that over half of ACSC admissions in children under five years of age were due to diarrhea and respiratory conditions ^9^.

The medium- and long-term impacts of state investment in health promotion, prevention, and PHC strengthening are well documented, with evidence demonstrating improvements in primary care efficiency, reductions in avoidable hospitalizations due to ACSC, and lower healthcare costs ^17^. However, trend analyses should also consider the impact of policies aimed at increasing investments in human resources, infrastructure, equipment, and medical supplies, as observed in Ecuador since 2010 ^18^.

Between 2000 and 2008, an upward trend in crude ACSC rates was observed. During this period, Ecuador adhered to neoliberal economic policies characterized by structural adjustment programs, marked by limited public healthcare investment and a reliance on out-of-pocket payments. In 2008, the new Constitution established the State as the guarantor of the right to health and positioned PHC as the foundation of the national health system, operationalized via the Comprehensive Healthcare Model and increased health investment ^19^. These reforms halted rises but maintained hospitalization rates.

Ecuador has progressively strengthened its immunization program, introducing new vaccines since 1999. The rotavirus vaccine was incorporated for infants under six months in 2007 ^20^, coinciding with a decline in hospital discharges due to gastrointestinal infections and complications, as indicated by the Joinpoint analysis (annual percent change - APC: -6.37%; p < 0.01). This suggests a potential beneficial impact of vaccination policies, despite the non-significant AAPC over the entire study period.

Since the onset of the COVID-19 pandemic in Ecuador in March 2020, healthcare services prioritized emergency response ^21^. Social isolation measures, mobility restrictions, and the shift to remote work disrupted access to healthcare, likely contributing to the overall decline in avoidable hospitalizations due to ACSC in 2020, and particularly due to respiratory diseases ^22^. In a cohort of Ecuadorian children with asthma, healthcare utilization declined sharply after the COVID-19 lockdown, while asthma exacerbations and inhaled corticosteroid use remained unchanged ^23^.

Our findings revealed an upward trend in hospitalizations due to skin and subcutaneous tissue infections over the study period, with a post-COVID-19 recovery. These results align with a global analysis (1990-2021) that reported increasing proportions across all age groups, with middle- to low-sociodemographic index regions being the most affected ^24^.

A 2021 study identified Ecuador as having the highest rates of hospitalizations for urinary tract infections worldwide ^25^. We also found increasing trends in urinary tract infections-related hospitalizations among children under five years of age. In contrast, recent evidence from Brazil reported a declining trend, underscoring the importance of early diagnosis via urine testing and timely treatment at the primary care level ^10^ ^,^ ^26^.

*Ecuador’s Ten-Year Health Plan 2022-2030* aims to strengthen the PHC-centered model, targeting a 10% reduction in avoidable hospitalizations due to ACSC rates across all age groups by 2030, using 2019 (15.5%) as the baseline year ^18^. In our study, the overall rate was 26% in 2019 and 23.3% in 2023, reflecting a cumulative reduction of 2.7% over four years. Forecasting predicted a reduction of approximately 6% by 2026. This goal is critical, as avoidable hospitalizations in children under five years of age accounts for a high proportion of ACSC, and no substantial decline has been observed beyond the temporary drop during the pandemic.

### Limitations

There are two limitations in our study: (1) potential ICD-10 misclassification and underreporting of diagnoses ^15^; and (2) forecast results should be interpreted with caution, as they assume the continuation of historical patterns and do not incorporate potential changes in health policies, health systems, or population demographics.

## Conclusion

Over the past 24 years, trends in crude avoidable hospitalizations due to ACSC rates among children under five years of age showed three distinct periods linked to macroeconomic policy shifts and the COVID-19 pandemic. Rates of respiratory diseases, urinary tract infections, and skin and subcutaneous tissue infections increased slightly, while gastrointestinal infections and complications was the only condition to decrease. Projections highlight the importance of evaluating current policies and their potential in achieving national goals. Future studies should investigate determinants of ACSC, especially in cantons where SMRs consistently exceed expected levels.

## Data Availability

The sources of information used in the study are indicated in the body of the article.
